# Chromosome level assembly of five *Brassica rapa* and *oleracea* accessions expand the resistance genes reservoir

**DOI:** 10.1038/s41597-025-06261-5

**Published:** 2025-12-11

**Authors:** C. Falentin, W. J. W. Thomas, M. Boudet, P. Le Boulch, A. Bourdais, L. Maillet, F. Legeai, K. Labadie, C. Cruaud, G. Deniot, J. Batley, A. Gravot, J. M. Aury, M. Rousseau-Gueutin

**Affiliations:** 1 IGEPP, INRAE, Institut Agro, University of Rennes, Le Rheu, 35653 France; 2 School of Biological Sciences, The University of Western Australia, Perth, WA Australia; 3 University of Rennes, INRIA, CNRS, IRISA, Rennes, France; 4 Génoscope, Institut François Jacob, CEA, CNRS, Université Evry, Université Paris-Saclay, 91057 Evry, France; 5 Génomique Métabolique, Genoscope, Institut François Jacob, CEA, CNRS, Université Evry, Université Paris-Saclay, 91057 Evry, France

**Keywords:** Plant evolution, Genome

## Abstract

*Brassica rapa* and *B. oleracea* are two diploid crops of agronomic importance, which display important phenotypic variation. They are the diploid progenitors of *B. napus* (oilseed rape), another major crop, which suffers from low genetic diversity. For that reason, *B. rapa* and *B. oleracea* are often used to introgress traits of interest into *B. napus*. In this study we assembled, at the chromosome level, the genomes of three *B. oleracea* accessions (including a wild type), and largely improved the genome assemblies of two *B. rapa* genotypes using Oxford Nanopore Technologies and Illumina sequencing. A total of 91.9 to 98.7% of the assembled sequences were anchored to pseudochromosomes. We also produced RNA-Seq data from different organs (flower bud, leaf, root and stem) for gene annotations. Overall, 94.97 to 99.49% of the predicted genes are on pseudomolecules. Finally, we also predicted their resistance gene analogs, including ones unique to each assembly. These five chromosome level assemblies represent a crucial resource to expand the known reservoir of disease resistance genes.

## Background & Summary

The *Brassica* genus contains several crop species cultivated as oilseeds, vegetables and condiments. It belongs to the Brassicaceae family, which comprises approximately 4,000 species and 350 genera^1,2^. *B. oleracea* and *B. rapa* are two agronomically important species that are primarily grown as vegetables. These two species diverged from a common ancestor about 4 million years ago. Both species present considerable phenotypic diversity which has been subjected to independent selection, giving rise to numerous morphotypes^3–6^. For example, different varieties of *B. oleracea* account for broccoli, brussels sprouts, cabbage, cauliflower and kale, among others, while *B. rapa* is grown as Chinese cabbage, pak choi, mizuna and turnip^5^.

*B. oleracea* and *B. rapa* are the diploid progenitors of *B. napus*, which was formed through their interspecific hybridization and genome doubling^7^. *B. napus*, also known as oilseed rape or canola, is one of the most economically important oilseed crops, processed into edible and industrial oil^8^. However, due to its polyploid origin and extensive human selection, primarily for seed quality traits, its genetic diversity has been severely eroded^9^. As a result, the diversity present in its parental diploid progenitors is often exploited and introgressed into *B. napus* to introduce agronomically favorable genes, including disease resistance (*R*) genes^10–13^.

With the recent advent of third-generation sequencing technologies, it is now possible to assemble plant genomes at the chromosome level, facilitating the identification and cloning of genes, including causal *R* genes, for example for blackleg^14,15^, clubroot^16^ and Sclerotinia stem rot^17^ in *Brassica*. However, the genome assembly of only a few individuals highlights the inadequacy of single reference genomes in capturing species-wide genetic diversity and therefore the requirement to construct pangenomes. Analysis of the *B. oleracea* and *B. rapa* pangenomes revealed that resistance gene analogs (RGAs) are highly affected by presence-absence variation, with 12% and 30%, respectively, forming the variable genomes of *B. oleracea* and *B. rapa*^18,19^. Having multiple genome assemblies which capture the diversity between accessions and morphotypes is therefore crucial in expanding the repertoire of known RGAs which underpins the identification of functional *R* genes.

Here, we report the construction of high-quality chromosome level genome assemblies for three *B. oleracea* accessions: *B. oleracea* ssp. *acephala* cv. C102, *B. oleracea* ssp. *botrytis* cv. Nd125, and a wild type *B. oleracea* individual ‘Bos01’ from Le Hode (Normandy, France). We also improved the genome assemblies of two previously published *B. rapa* accessions: *B. rapa* ssp. *narinosa* cv. Wutacai^4^ and *B. rapa* ssp. *trilocularis* cv. R500^20^. When compared with previous versions of the same accession or other accessions of the same morphotype, our assemblies contain, on average, over 13,000 additional gene annotations which include novel RGAs. These assemblies provide a valuable resource for the exploration of novel RGAs which have the potential to contribute toward the improvement of disease resistance in *Brassica* crops.

## Methods

### Plant material, DNA extraction, sequencing

One individual of two *B. rapa* (*B. rapa* ssp*. narinosa* cv. Wutacai and *B. rapa* ssp*. trilocularis* cv. R500) and three *B. oleracea* accessions (*B. oleracea* ssp*. acephala* cv C102, *B. oleracea* ssp*. botrytis* cv Nd125, a wild type *B. oleracea* ‘Bos01’ individual from Le Hode, Normandy, France) were grown in a greenhouse (16 h of light at 21 °C followed by 8 h of dark at 18 °C). Plants were grown in pots filled with a non fertilized commercial substrate (Falienor, reference 922016F3) and irrigated twice a week with a commercial fertilized solution (Liquoplant Blue, 2.5% nitrogen, 5% phosphorus, 2.5% potassium, w/v). These different accessions were retrieved from the BraCySol Biological Resource Center (https://eng-igepp.rennes.hub.inrae.fr/about-igepp/platforms/bracysol). The collected plant materials were flash frozen and stored at −80 °C. High-quality high-molecular weight (HMW) DNA was generated for each accession from 1 g of young leaves of a single individual using a CTAB extraction followed by a purification using the commercial Qiagen Genomic-tip (QIAGEN, Germantown, MD, USA), as previously described^21^. HMW gDNA quality was checked on a FemtoPulse system (Agilent), revealing DNA molecules to be over 40 kb. For each accession, a library was prepared using the Native Barcoding Kit 24 V14 - Ligation sequencing gDNA (SQK-NBD114.24). HMW DNA libraries were sequenced on PromethION flow cells. In addition, Illumina DNA sequencing was performed using a NovaSeq 6000 (2*150 paired-end reads). Raw DNA Seq data are available on ENA: PRJEB91561 (*B. oleracea* ‘Bos01’), PRJEB91565 (*B. oleracea* cv. C102), PRJEB91569 (*B. oleracea* cv. Nd125), PRJEB91574 (*B. rapa* cv. R500), PRJEB91578 (*B. rapa* cv. Wutacai)^22^.

### *De novo* assemblies of chromosome level nuclear genomes

The two *B. rapa* and three *B. oleracea* nuclear genomes were assembled using the Genoscope GALOP pipeline (https://workflowhub.eu/workflows/1200*)*. Briefly, raw Nanopore reads were assembled using NextDenovo v2.5.1 (Nextomics, https://github.com/Nextomics/NextDenovo). The resulting contigs were first polished with Medaka v1.7.2 (https://github.com/nanoporetech/medaka) using default parameters and Nanopore long reads. These contigs were then further polished with two rounds of Hapo-G v1.1^23^, using Illumina short reads and default parameters. They were finally scaffolded using Ragtag v2.1.0^24^, with either the *B. rapa* Z1 v2^25^ or the *B. oleracea* cv. Korso genome^26^ as the reference, depending on the species. A schematic diagram summarizing the workflow used for *de novo* assembly of the nuclear genomes is presented in Figure S1.

### RNA extraction, sequencing and gene prediction

To aid gene prediction, Illumina RNA-Seq data were obtained for each accession using different organs that were harvested on the same plant (same as the one used for DNA sequencing) at different developmental stages. More precisely, we harvested leaves, roots and stems on plants at the 4–6 leaf stage, and flower buds on mature plants. The different organs were first harvested separately and flash frozen. They were then ground into a fine powder using a mortar and pestle. A similar quantity of powder from the different organs was bulked and used to extract total RNA using the Nucleospin RNA Plus kit (Macherey-Nagel, Germany). The cDNA library was constructed using NEBNext® Ultra™ RNA Library Prep kit for Illumina (New England Biolabs, USA) and Illumina paired-end sequencing was performed on a Illumina NovaSeq 6000 (Azenta Life Sciences, Germany). Raw RNA Seq data are available on ENA: PRJEB91561 (*B. oleracea* ‘Bos01’), PRJEB91565 (*B. oleracea* cv. C102), PRJEB91569 (*B. oleracea* cv. Nd125), PRJEB91574 (*B. rapa* cv. R500), PRJEB91578 (*B. rapa* cv. Wutacai)^22^.

Gene prediction was performed using several reference proteomes: eight from other *B. napus* genotypes (Westar, ZS11, Quinta, Zheyou7, No2127, Gangan, Tapidor and Shengli)^27^; *Arabidopsis thaliana* (proteome ID: UP000006548); *B. rapa* cv. Z1^25^; *B. oleracea* cv. HDEM^28^; and *B. napus* cv Darmor-bzh^29^. Regions of low complexity in the genomic sequences were masked using the DustMasker algorithm (version 1.0.0 from the BLAST + 2.10.0 package)^30^. Protein sequences were aligned to the genome using a two-step strategy. First, BLAT v36^31^ was used to rapidly localize putative matches. The best hit and all hits with a score ≥ 90% of the best match were retained. In the second step, alignments were refined using Genewise v2.2.0^32^, which accurately identifies intron-exon boundaries. Alignments were retained if more than 75% of the protein length aligned to the genome. Additionally, RNA-Seq short reads (Illumina) were used for four genomes (R500, Wutacai, C102, and Nd125). Reads were mapped to their respective genomes using HISAT2 v2.2.1^33^ with default parameters. The resulting BAM files were used as an input in StringTie v2.2.3^34^, with the–rf option to indicate the orientation of the RNA-Seq libraries. When multiple transcripts were detected for a gene, the most highly expressed one (based on TPM) was selected. GFF files were derived from StringTie outputs to retain only the most highly expressed transcript and to remove single-exon models.

All transcriptomic and protein alignments were integrated using Gmove (https://f1000research.com/posters/5-681), an evidence-driven gene predictor requiring no training. Gmove constructs a graph where nodes and edges represent putative exons and introns extracted from alignments, then extracts paths consistent with the protein evidence, identifying open reading frames. Predicted gene models with more than 50% Untranslated Transcribed Region (UTR) content and with a coding sequence (CDS) length shorter than 300 nucleotides were discarded. All final gene models were renamed according to the MBGP (Multinational Brassica Genome Project) nomenclature. A schematic diagram summarizing the workflow used for *de novo* assembly of the nuclear genomes is presented in Figure S2. The annotations of the nuclear genes (.gff, mRNA and protein files) are available on the French recherche.data.gouv repository: 10.57745/D21PQM^35^ (deposited October 2025).

The quality of the genomes was also evaluated based on their Long Tandem Repeat (LTR) composition using LAI version beta3.2^36^. The LTR annotation was performed with LTR_retriever version 3.0.4^37^, which integrated results from both ltrharvest^38^ (GenomeTools version 1.6.2, using the following parameters: -minlenltr 100, -maxlenltr 7000, -mintsd 4, -maxtsd 6, -motif TGCA, -motifmis 1, -similar 85, -vic 10, -seed 20, -seqids yes) and LTR_finder^39^ (parallel version 1.3, with the options -harvest_out and -size 1000000). This process followed the recommandations provided at https://github.com/oushujun/LTR_retriever.

### Chloroplast genome assemblies

The Illumina DNA Seq data obtained for each accession were also used to assemble the chloroplast genomes of each accession. This was performed using FastPlast v1.2.9^40^ (https://github.com/mrmckain/Fast-Plast). The chloroplast genome assemblies were annotated using the online version of GeSeq^41^. These assembled and annotated genomes were then validated visually using Geneious Prime 2022.2.2 and the *Arabidopsis thaliana* chloroplast genome (NC_000932)^42^ as a reference. A graphical representation of these different chloroplast genomes was obtained using the online OGDRAW v1.3.1 (https://chlorobox.mpimp-golm.mpg.de/OGDraw.html)^43^. Genome assemblies are available on ENA: PRJEB91562 (*B. oleracea* ‘Bos01’), PRJEB91566 (*B. oleracea* cv. C102, PRJEB91570 (*B. oleracea* cv. Nd125), PRJEB91575 (*B. rapa* cv. R500), PRJEB91579 (*B. rapa* cv. Wutacai)^22^. The annotations of the chloroplast genes (.gff) are available on the French recherche.data.gouv repository: 10.57745/RLHXJH^44^ (deposited October 2025).

## Data Records

All sequencing data and associated materials are available in the European Nucleotide Archive (ENA) under project accession number PRJEB91446^22^. The identifiers for the raw DNA and RNA-Seq data are: PRJEB91561 (*B. oleracea* ‘Bos01’), PRJEB91565 (*B. oleracea* cv. C102), PRJEB91569 (*B. oleracea* cv. Nd125), PRJEB91574 (*B. rapa* cv. R500), PRJEB91578 (*B. rapa* cv. Wutacai). The identifiers for the chloroplast genome assemblies are: PRJEB91562 (*B. oleracea* ‘Bos01’), PRJEB91566 (*B. oleracea* cv. C102), PRJEB91570 (*B. oleracea* cv. Nd125), PRJEB91575 (*B. rapa* cv. R500), PRJEB91579 (*B. rapa* cv. Wutacai). For each studied genotype, the accessions and their related bioprojects (DNA and RNA-Seq raw data, as well as nuclear and chloroplast genome assemblies) are summarized in Table S1. The annotations of the nuclear (.gff, mRNA and protein fasta files) are available on the French recherche data.gouv repository at 10.57745/D21PQM^35^ (deposited October 2025). On the same repository, the annotations of the chloroplast (.gff) genomes are available at 10.57745/RLHXJH^44^ (deposited October 2025).

## Technical Validation

### Evaluation of the ***de novo*** assembled genomes

**Fig. 1:**
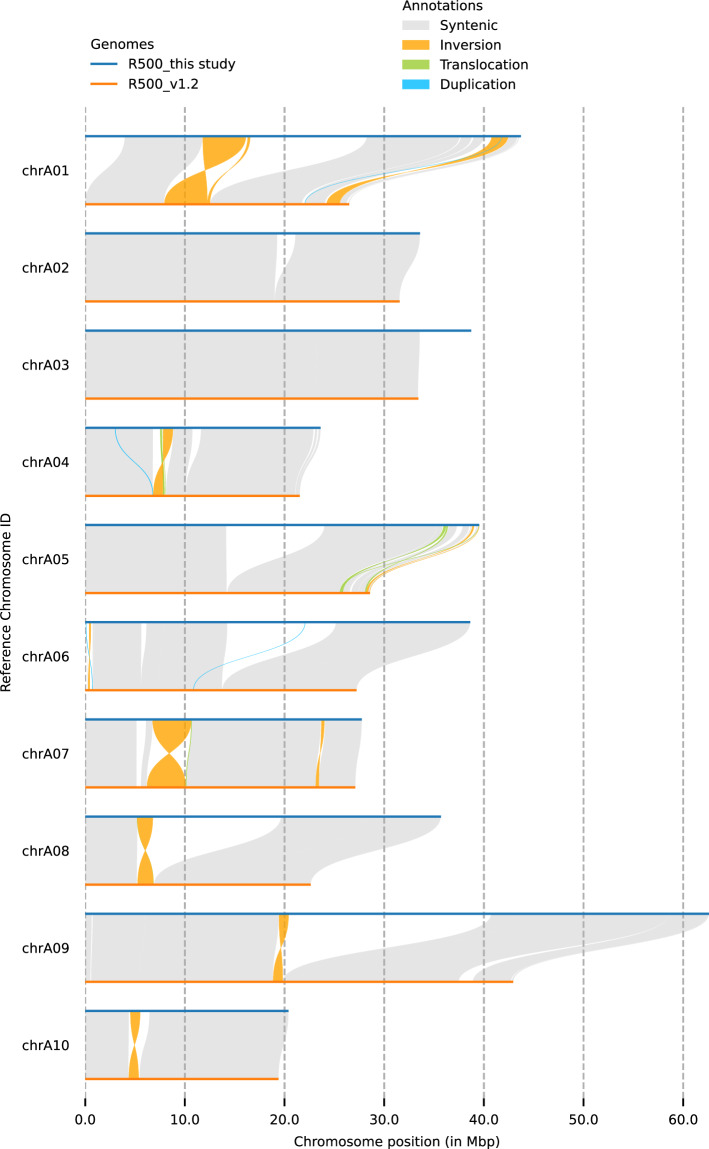
Comparison using SyRI^45^ of the new (this study) and previously published^20^ *B. rapa* ssp*. trilocularis* cv. R500 pseudomolecules.

For all five accessions, we obtained genome assemblies ranging from 563 to 646 Mb and from 373 to 404 Mb in *B. oleracea* and *B. rapa*, respectively. Overall, 91.9 to 98.7% of the sequences were anchored to pseudomolecules. For the two *B. rapa* accessions, for which a nuclear genome assembly was previously published^4,20^, our assemblies are far more complete than the previous versions (Table 1), as exemplified from the pseudomolecule size. For the *B. rapa* R500 genome, for which there was a genome assembly at the chromosome level, we used SyRI v1.5.4^45^ to identify the regions that were better assembled in our genome assembly and observed that our updated version was particularly improved in the highly repetitive pericentromeric regions, but also at the beginning of chromosome A01 (Fig. 1). For *B. oleracea*, we also compared the metrics of our genomes to those obtained in other accessions belonging to the same morphotype (Table 1). The genomes used for these analyses were the following: C-8 v2^46^; Korso v1^47^; 07-DH-33 v1 and W1701 v1^26^; T09 v1, T10 v1, T18 v1, T21 v1 and T25 v1^48^; and W03 v1^49^. For these comparisons, the metrics were not extracted from the original publication (except for R500 as indicated in Table 1) but were rather calculated from the downloaded FASTA files using an internal tool named fastoche (https://github.com/institut-de-genomique/fastoche). For the *B. oleracea* genomes, 91.9 to 98.7% of the assembled sequences were anchored to pseudochromosomes.

**Table 1:** Summary of genome assembly metrics for the five new assemblies reported in this study and previous assemblies of the same accession (*B. rapa*) or different accessions of the same morphotype (*B. oleracea*).

Quality metric	*Brassica rapa*				*Brassica oleracea*												
	ssp. *trilocularis*		ssp. *narinosa*		ssp. *acephala*			ssp. *botrytis*					ssp. *oleracea*				
	R500 (this study)	R500 (ref. ^20^)	Wutacai (this study)	Wutacai (ref. ^4^)	C102 (this study)	07-DH-33 (ref. ^26^)	T18 (ref. ^48^)	Nd125 (this study)	T21 (ref. ^48^)	T25 (ref. ^48^)	Korso (ref. ^47^)	C-8 (ref. ^46^)	Bos01 (this study)	T09 (ref. ^48^)	T10 (ref. ^48^)	W03 (ref. ^49^)	W1701 (ref. ^26^)
Cumulative size (bp)	373,325,180	356, 000, 000 (a)	404,489,077	466,502,319	563,329,934	588,360,242	553,363,182	567,916,392	534,419,652	547,890,678	552,847,295	568,515,523	646,170,971	576,023,240	580,974,251	630,729,510	634,424,806
Sequences	33	1,753 (a)	80	12,558	31	10	26	88	72	138	10	10	157	15	24	360	11
Pseudomolecules	10	10	10	0	9	9	9	9	9	9	9	9	9	9	9	9	9
Pseudomolecule size (bp) (% of assembly)	364,236,165 (97.6)	280,850,083 (78.8)	375,103,728 (92.7)	N/A	555,962,841 (98.7)	588,287,377 (99.9)	550,420,920 (99.5)	552,818,513 (97.3)	526,630,535 (98.5)	524,691,661 (95.8)	547,555,460 (99.6)	557,113,381 (98.0)	594,003,544 (91.9)	572,017,529 (99.3)	570,456,620 (98.2)	594,346,218 (94.2)	618,795,957 (97.5)
Scaffold N50 (bp)	38,623,307	N/A	43,704,723	907,768	61,343,825	64,701,984	65,433,663	62,575,981	56,484,001	57,349,439	59,208,503	65,683,070	62,410,918	60,668,869	68,709,032	64,564,754	72,197,880
Scaffold N90 (bp)	23,616,232	N/A	21,064,187	10,792	51,763,739	58,208,169	54,329,710	47,990,424	49,547,750	46,904,723	52,701,841	48,937,047	54,501,227	56,592,305	50,417,254	49,313,063	53,632,606
Longest scaffold (bp)	62,577,379	24,500,000 (a)	62,011,682	9,177,230	81,909,429	80,057,606	78,331,576	75,538,215	74,621,860	73,465,279	78,655,145	79,842,669	87,029,299	78,617,262	81,748,926	80,735,250	86,880,579
Contig N50 (bp)	13,023,702	NA	11,339,266	793.238	28,762,279	11,430,835	20,808,756	12,119,172	5,552,566	11,427,368	8,222,774	21,552,297	7,307,935	2,5081,548	25,739,047	2,305,478	15,574,327
GC content (%)	36.757	29.205	37.417	38.353	36.802	36.995	36.737	36.702	36.635	36.141	36.502	36.144	36.903	36.891	37.037	36.725	37.255
LTR Assembly Index	12.13	-	10.19	5.91	14.74	6.98	6.95	14.79	7.90	6.52	13.91	5.85	12.81	5.77	6.95	14.0	4.16
BUSCO (6.0.0) scores brassicales odb12 (2025-07-01) (N = 4,311)	C:99.3% D:41.2% F:0.0% M:0.7%	C:97.3% D:39.3% F:0.0% M:2.7%	C:99.3% D:42.2% F:0.0% M:0.7%	C:99.4% D:47.2% F:0.0% M:0.6%	C:99.4% D:42.5% F:0.1% M:0.6%	C:99.3% D:42.5% F:0.1% M:0.6%	C:99.1% D:42.6% F:0.1% M:0.8%	C:99.4% D:44.3% F:0.0% M:0.6%	C:99.3% D:42.4% F:0.0% M:0.7%	C:99.2% D:42.5% F:0.0% M:0.7%	C:99.2% D:42.2% F:0.1% M:0.7%	C:99.4% D:42.5% F:0.1% M:0.6%	C:99.3% D:48.3% F:0.1% M:0.6%	C:99.3% D:42.4% F:0.1% M:0.7%	C:99.3% D:42.8% F:0.1% M:0.6%	C:99.3% D:49.8% F:0.0% M:0.7%	C:99.4% D:43.1% F:0.1% M:0.6%
Protein-coding genes	52,275	45,538	54,266	47,602	66,049	52,913	53,089	68,060	51,515	50,754	60,640	57,983	71,867	52,057	52,606	62,059	51,831
Genes within pseudo-molecules	51,796	45,538	52,977	N/A	65,713	52,909	53,072	66,432	51,464	50,541	60,027	57,189	68,253	52,001	52,581	60,900	51,586

(a) These values were inferred from ref. ^20^ as the scaffolds are not provided in the published fasta file.

Taking advantage of RNA-Seq data obtained from various organs for each accession, we predicted protein-coding genes, and observed 66,049 to 71,867 and 52,275 to 54,266 protein coding sequences in our *B. oleracea* and *B. rapa* genotypes, respectively. Importantly, 94.97 to 99.49% of these predicted genes are anchored on pseudomolecules.

Our five newly assembled nuclear genomes were also validated by launching BUSCO v5.8.2^50^ with the brassicales_odb12 dataset (July 2025), revealing a gene completeness of 99.3 to 99.4%. The high quality of our genome assemblies can also be observed through the values obtained for the LTR Assembly Index (value over 10, as expected for reference quality genomes). There was no score for the first genome assembly of *B. rapa* cv. R500^20^ as the total and intact LTR sequence content was too low in this first draft assembly for accurate LAI calculation.

**Fig. 2:**
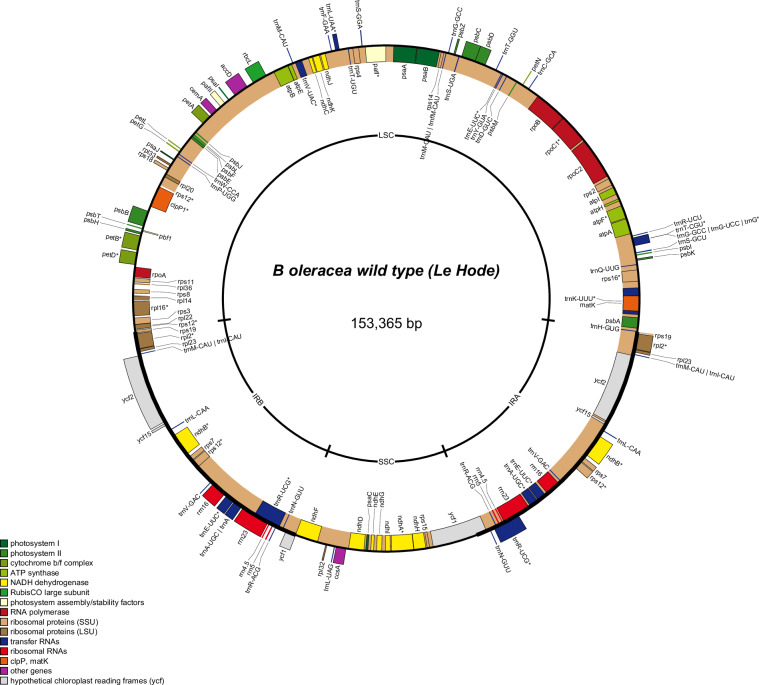
Graphical representation of the wild *B. oleracea ‘*Bos01’ assembled and annotated chloroplast genome.

Using the Illumina DNA-Seq data obtained for our five genotypes, we also assembled, annotated, and graphically represented their chloroplast genomes using FastPlast v1.2.8^40^, as well as GeSeq^41^ and OGDRAW^43^ online versions (example in Fig. 2). Their size ranged from 153,364 to 153,365 bp and from 153,036 to 153,464 bp in *B. oleracea* and *B. rapa*, respectively.

### Evaluating and expanding the lists of resistance gene analogs

**Fig. 3:**
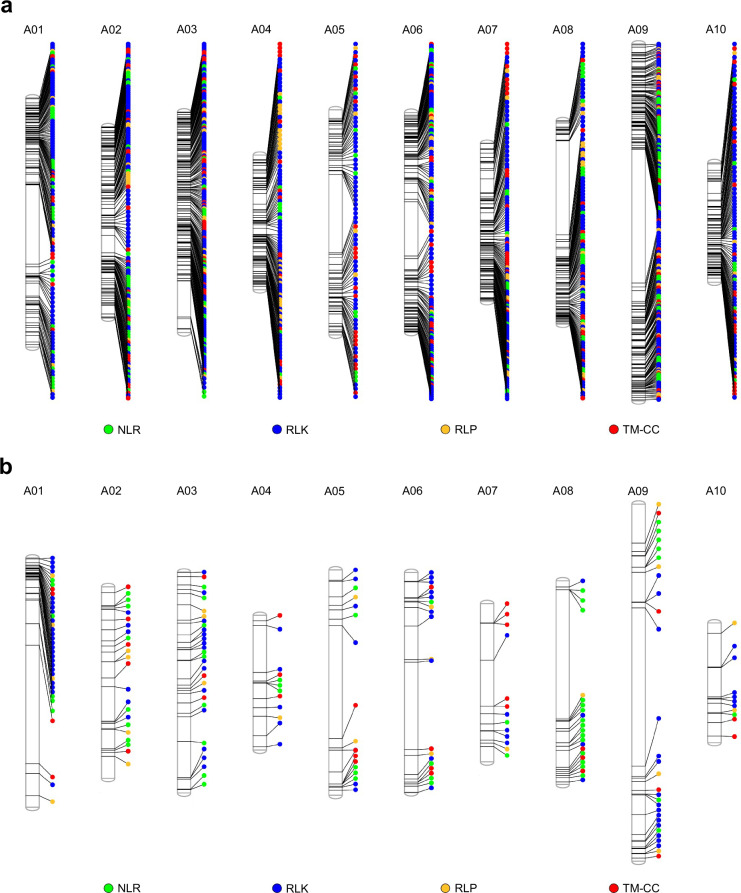
The distribution of resistance gene analogs (RGAs) across the new (this study) *B. rapa* ssp. *trilocularis* cv. R500 pseudomolecules. The plots includes (**a**) all RGAs or (**b**) RGAs that are unique when compared with the previously published^20^ R500 assembly.

To evaluate the quality of our nuclear genome assemblies and their utility compared to previous versions or assemblies from other accessions belonging to the same morphotype, we explored their RGA content using RGAugury v2.1.7m^51^. We predicted 1,382 to 1,411 and 1,703 to 1,912 RGAs in our *B. rapa* and *B. oleracea* genotypes, respectively (Table 2, see 10.57745/O2FUQ8 for a list of RGA genes identified in each genome). To explore the variability of the RGA content between assemblies, we used OrthoFinder v3.0.1^52^. OrthoFinder analysis identified at least 122 novel RGAs in Nd125, Bos01 and C102, when compared to other accessions from the same morphotype (Table 3, orthogroups from these analyses can been obtained at 10.57745/O2FUQ8^53^). Additionally, over 200 novel RGAs were identified in both Wutacai and R500 (this study) when compared to their previous versions (Table 3, lists of all the unique RGAs for each newly assembled genome are available at 10.57745/O2FUQ8^53^). For the new R500 assembly, we graphically represented using phenogram^54^ the distribution of the different types of RGAs along each chromosome, allowing the identification of RGA clusters at different chromosomic regions (Fig. 3a). We also only graphically represented the newly identified RGAs in R500, highlighting that these genes are distributed on all chromosomes but are overrepresented at the beginning of chromosome A01 (Fig. 3b), which was not assembled in the previous version (Fig. 1).

**Table 2:** Number and class of resistance gene analogs (RGAs) predicted in the five new genomes assembled in this study and comparisons with previous versions of the same accession (*B. rapa*) or different accessions of the same morphotype (*B. oleracea*).

Species	Subspecies	Accession	NLR								TM-LRR			Total
			CN	CNL	NBS	NL	OTHER	TN	TNL	TX	RLK	RLP	TM-CC	
*Brassica rapa*	*trilocularis*	R500 (this study)	6	27	20	26	12	19	75	53	780	137	227	1,382
	*trilocularis*	R500 (ref. ^20^)	7	29	15	41	8	3	30	34	710	123	259	1,259
	*narinosa*	Wutacai (this study)	5	32	14	30	18	18	75	50	792	130	247	1,411
	*narinosa*	Wutacai (ref. ^4^)	6	12	11	29	5	8	20	36	727	124	246	1,224
*Brassica oleracea*	*acephala*	C102 (this study)	14	41	34	55	21	46	112	99	866	148	267	1,703
	*acephala*	07-DH-33 (ref. ^26^)	10	28	23	48	15	20	62	84	714	112	225	1,341
	*acephala*	T18 (ref. ^48^)	7	17	15	39	13	14	54	47	682	133	246	1,267
	*botrytis*	Nd125 (this study)	14	49	39	58	23	41	114	103	872	166	272	1,751
	*botrytis*	T21 (ref. ^48^)	8	20	20	56	6	13	32	40	610	90	260	1,155
	*botrytis*	T25 (ref. ^48^)	7	9	5	25	12	22	52	159	605	78	241	1,215
	*botrytis*	Korso (ref. ^47^)	19	36	38	64	10	21	78	72	708	90	237	1,373
	*botrytis*	C8 (ref. ^46^)	7	9	12	28	12	27	64	47	614	103	228	1,151
	*oleracea*	Bos01 (this study)	12	52	37	55	28	44	124	105	969	195	291	1,912
	*oleracea*	T09 (ref. ^48^)	9	19	25	43	11	12	44	41	530	100	244	1,078
	*oleracea*	T10 (ref. ^48^)	7	11	18	36	13	15	36	50	757	112	258	1,313
	*oleracea*	W03 (ref. ^49^)	10	22	41	39	16	35	78	92	731	93	229	1,386
	*oleracea*	W1701 (ref. ^26^)	10	29	21	35	13	17	61	86	755	95	237	1,359

**Table 3:** The number of unique resistance gene analogs (RGAs) in the five new genome assemblies when compared to previous versions of the same accession (*B. rapa*) or different accessions of the same morphotype (*B. oleracea*).

Species	Subspecies	Reference assembly	Assemblies compared	Number of RGAs unique to reference
*Brassica oleracea*	*acephala*	C102 (this study)	07-DH-33 (ref. ^26^), T18 (ref. ^48^)	254
	*botrytis*	Nd125 (this study)	T21 (ref. ^48^), T25 (ref. ^48^), Korso (ref. ^47^), C8 (ref. ^46^)	122
	*oleracea*	Bos01 (this study)	T09 (ref. ^48^), T10 (ref. ^48^), W03 (ref. ^49^), W1701 (ref. ^26^)	142
*Brassica rapa*	*trilocularis*	R500 (this study)	R500 v1.2 (ref. ^20^)	219
	*narinosa*	Wutacai (this study)	Wutacai (ref. ^4^)	256

## Supplementary information

Supplementary Figures

Table S1

## Data Availability

All raw DNA and RNA sequencing data are available in the European Nucleotide Archive (ENA) under project accession number PRJEB91446: PRJEB91561 (*B. oleracea* ‘Bos01’), PRJEB91565 (*B. oleracea* cv. C102), PRJEB91569 (*B. oleracea* cv. Nd125), PRJEB91574 (*B. rapa* cv. R500), PRJEB91578 (*B. rapa* cv. Wutacai). The nuclear and chloroplast genomes are also available on ENA: PRJEB91562 (*B. oleracea* ‘Bos01’), PRJEB91566 (*B. oleracea* cv. C102, PRJEB91570 (*B. oleracea* cv. Nd125), PRJEB91575 (*B. rapa* cv. R500), PRJEB91579 (*B. rapa* cv. Wutacai). The numbers for the different accessions and their related bioprojects (DNA and RNA-Seq raw data, as well as nuclear and chloroplast genome assemblies) are given in the supplementary Table S1. The annotations of the nuclear (.gff, mRNA and protein fasta files) are available on the French recherche.data.gouv repository at 10.57745/D21PQM^35^. On the same repository, the annotations of the chloroplast (.gff) genomes are available at 10.57745/RLHXJH^44^. We also provided tabular files (10.57745/O2FUQ8^53^, October 2025) giving: (i) the gene names of the Resistance Gene Analogs identified in all genome assemblies generated in this study and those used for comparisons, (ii) the RGA genes that were unique to our newly assembled genotype compared to others from the same morphotype (*B. oleracea* cv. C102; *B. oleracea* cv. Nd125; *B. oleracea* ‘Bos01’) or from previous genome assemblies (*B. rapa* cv. R500; *B. rapa* cv. Wutacai); (iii) the orthologous relationships of RGAs between our newly assembled genotypes and those from the same morphotype or from the previously assembled genome (*B. oleracea* cv. C102; *B. oleracea* cv. Nd125; *B. oleracea* ‘Bos01’; *B. rapa* cv. R500; *B. rapa* cv. Wutacai). For all these comparisons, the previously published assembled genomes were the following for *B*. oleracea: C-8 v2^46^; Korso v1^47^, 07-DH-33 v1 and W1701 v1^26^; T09 v1, T10 v1, T18 v1, T21 v1 and T25 v1^48^; and W03 v1^49^; for *B. rapa*: Wutacai^4^, R500^20^.

## Code availability

This study did not produce new codes but rather used publicly available codes. All data analyses were performed using bioinformatic tools. The software versions and used parameters are detailed in the Methods section. When not specified, default parameters were applied.
