# Supplementary figures and images for: Chromosome level assembly of five Brassica rapa and oleracea accessions expand the resistance genes reservoir
Source: Sci Data. 2025 Dec 11;12:2016. doi: 10.1038/s41597-025-06261-5 (PMC12749581; doi:10.1038/s41597-025-06261-5)

# Figure S1: Genome assembly process

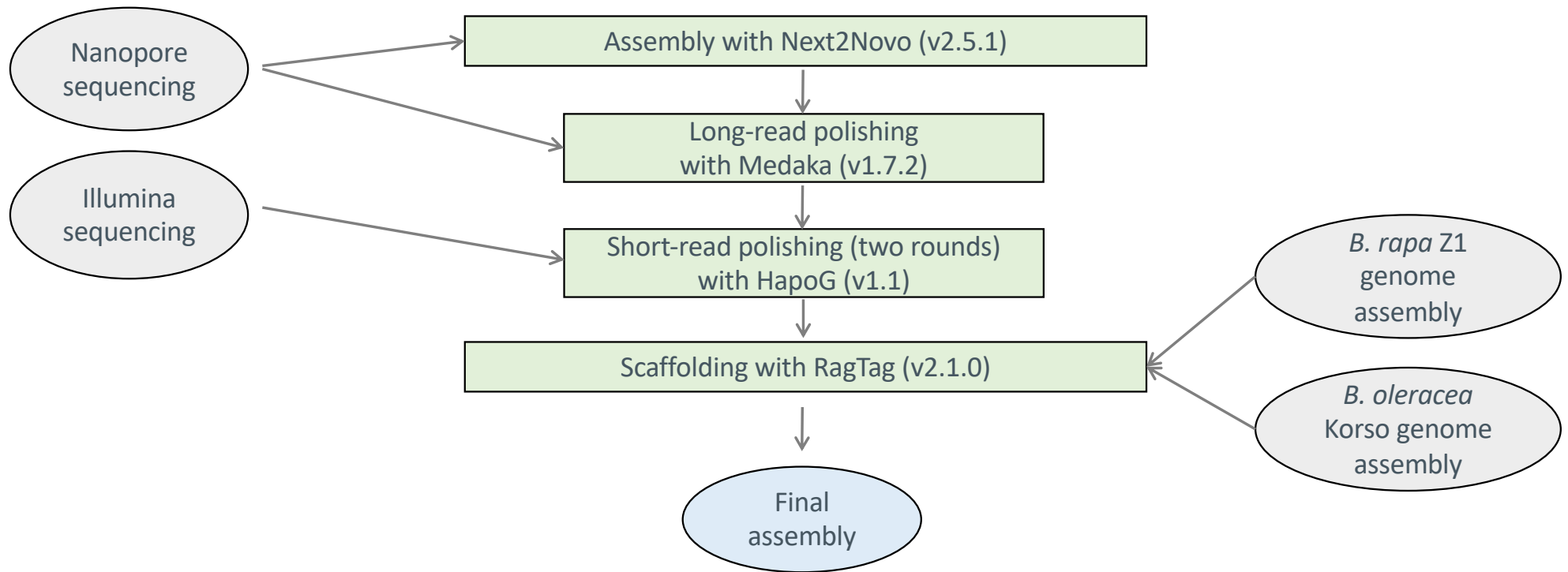

# Figure S2: Genome annotation process

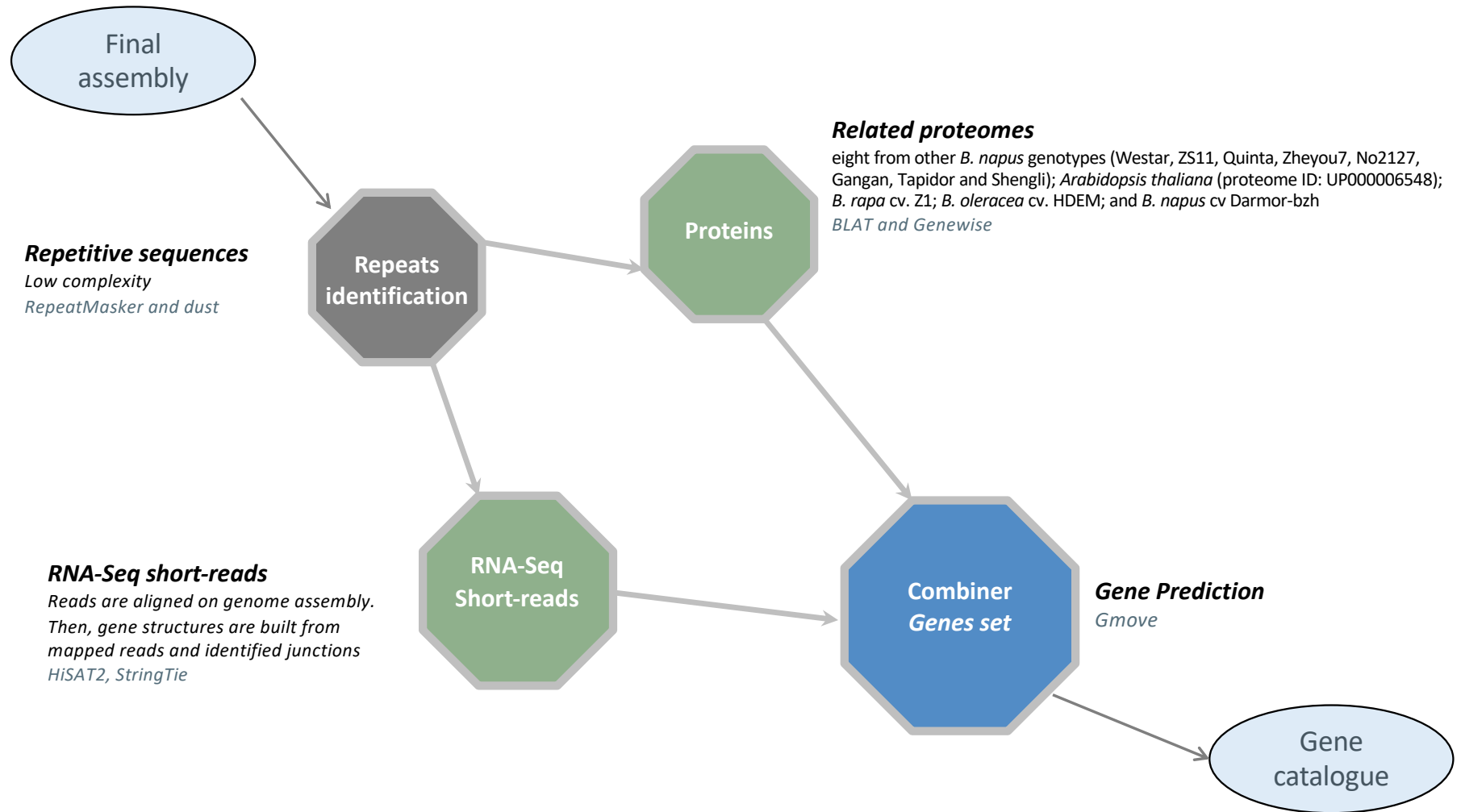

Supplement: Supplementary file 1 — Supplementary Figures [file 41597_2025_6261_MOESM1_ESM.pdf]
